# Shortened outreach periodontal therapy in nursing home residents with periodontitis: A randomized controlled trial

**DOI:** 10.1002/jper.70041

**Published:** 2025-12-26

**Authors:** Anna G. Barbe, Dalia Beck, Martin Hellmich, Max von Kohout, Sonja H. M. Derman, Dirk Bleiel

**Affiliations:** ^1^ Polyclinic for Operative Dentistry and Periodontology Faculty of Medicine and University Hospital Cologne, University of Cologne Cologne Germany; ^2^ Institute of Medical Statistics and Computational Biology (IMSB) University of Cologne, Faculty of Medicine and University Hospital Cologne Faculty of Medicine and University Hospital Cologne, University of Cologne Köln Germany; ^3^ Department of Medical Statistics University Medical Center Göttingen, Georg‐August‐University Göttingen Germany; ^4^ Joint Dental Practice, Dres. Pia und Dirk Bleiel Rheinbreitbach Germany

**Keywords:** dental plaque, gingivitis, nursing homes, periodontitis, randomized controlled trial, subgingival curettage

## Abstract

**Background:**

We examined the clinical effectiveness of shortened outreach periodontal therapy for 3 months in nursing home residents with periodontitis versus controls (no therapy).

**Methods:**

Nursing home residents with periodontitis were randomly assigned to either (a) one‐time, shortened outreach periodontal therapy adapted to German statutory health guidelines for periodontitis patients in need of care, which included subgingival instrumentation as part of anti‐infective therapy (intervention) or (b) no treatment (control). Changes in bleeding on probing (BOP), plaque index (PI), and pocket probing depth (PPD) were assessed before therapy and at 3 months.

**Results:**

Thirty‐six participants were included (mean age 85 ± 6 years). In the intervention group, BOP decreased longitudinally from baseline (45 ± 20) to follow‐up (35 ± 16) (change (∆) −10 ± 26; *p* = 0.088; effect size of Cohen's *d* = −0.70 (95% confidence interval (CI) [−1.40 to 0.00]). Significant decreases were observed for PI (from 1.0 ± 0.4 to 0.7 ± 0.4, respectively; ∆−0.3 ± 0.4; *p* = 0.010; Cohen's *d* = −0.56 (95% CI [−1.24 to 0.11]) and PPD (4.5 ± 0.5 to 4.0 ± 0.5 mm; ∆−0.5 ± 0.5; *p* = 0.004; Cohen's *d* = −1.32; 95% CI [−2.05 to −0.59]). Between‐group comparisons revealed significant differences in BOP (*p* = 0.041) and PPD (*p* < 0.001), but not PI (*p* = 0.09). In generalized linear modeling, the treatment group was the main influencing factor, with oral or vestibular plaque localization also affecting treatment outcomes over time.

**Conclusion:**

In nursing home residents with high plaque levels and periodontal burden, shortened outreach periodontal therapy yielded improvements in periodontal health among nursing home residents, though limited by insufficient oral hygiene and the population's high vulnerability. Trial registration: DRKS database (no. DRKS00029392).

**Clinical trial registration:**

German Clinical Trials Register (DRKS), registration number DRKS00029392.

**Plain language summary:**

This controlled study looked at the effectiveness of a special dental treatment for elderly nursing home residents with gum disease (periodontitis). The treatment, which involved cleaning and instrumentation below the gum line, was compared to no treatment. Thirty‐six participants (aged 85 years, on average) were included in the study. The results showed that after 3 months, the treatment group had a significant reduction in signs of gum inflammation (measured by bleeding on probing) and a decrease in the depth of the pockets around the teeth—both indicators of gum disease. These improvements were not seen in the control group, which did not receive the treatment. The plaque levels in the mouth decreased slightly in the treatment group but did not show a strong difference compared to the control group. Overall, the findings suggest that even for older adults in nursing homes, a simple dental procedure to clean beneath the gums can improve gum health, reduce inflammation, and lower the risk of further gum disease. However, the improvements were modest, likely due to difficulties in maintaining adequate daily oral hygiene within this vulnerable population. This underscores both the importance of providing dental and periodontal care in nursing homes and the need for enhanced support with daily oral hygiene to achieve satisfactory outcomes.

## 1 INTRODUCTION

In Western countries, an increasing number of older adults retain their natural teeth, with forecasts predicting a further increase over the coming decades.^1^, ^2^, ^3^, ^4^ This presents significant challenges in dental and periodontal care, especially among elderly people who need care in institutionalized settings where dental care is limited, for example, by factors such as multimorbidity and reimbursement of dental services. A systematic review has highlighted that nearly all study participants in nursing homes suffered from gingivitis, and most had moderate‐to‐severe periodontitis with significant attachment loss.^5^ This alarming trend was assessed using various periodontal indices, including those designed specifically for these populations (the Plaque Index/Gingival Index for Long‐Term Care^6^) and more traditional indices as the Community Periodontal Index of Treatment Needs (CPITN)).^7^, ^8^

The high burden of periodontitis among older individuals is evident across various studies. In one, 40.7% of people aged ≥65 years had attachment loss ≥6 mm, while 22.7% had probing pocket depths of ≥5 mm.^9^ German epidemiological data indicate similar results: > 90% of people requiring care suffer from moderate‐to‐severe periodontitis,^10^ reflecting the immense need for outreach dental care in this population. Studies also indicate a strong association between dementia and periodontal disease, with periodontal pockets ≥4 mm evident in 73.8% of patients with dementia.^11^

Poor oral health among older people causes direct pain and adverse health outcomes, including malnutrition, weight loss, speech difficulties, increased risk of aspiration pneumonia and stroke, and compromised immune function and metabolic control in diabetes.^12^, ^13^, ^14^ There are also indications that tooth loss is associated with a higher risk of dementia;^15^, ^16^ the impact of periodontal disease on nutrition and malnutrition is a significant risk factor for frailty.^17^ Frailty itself is a state of reduced physiological reserve and increased vulnerability to stressors, leading to adverse health outcomes such as dependency, cognitive decline, and mortality.^18^, ^19^ Recent data demonstrate that severe periodontal disease is associated with all‐cause and cause‐specific mortality among US adults.^20^

Beyond the direct clinical and systemic implications, poor oral health has substantial health economic ramifications. Improved oral health among older populations can reduce the incidence of non‐ventilator‐associated hospital‐acquired pneumonia, potentially leading to significant cost savings.^21^ But dental care utilization among those in need of care is reduced, suggesting that outreach services for this group require improvement.^22^, ^23^, ^24^

The provision and implementation of adequate periodontal therapy for elderly people in need of care in nursing homes is a task that dentistry will increasingly encounter in the future. In Germany, the German Social Code (§22a Sozialgesetzbuch [SGB] V) and the periodontitis (PAR) guidelines of the statutory health insurance (Gesetzliche Krankenversicherung [GKV]) outline different approaches to periodontitis therapy, mainly concerning outreach care and systematic periodontitis treatment in the practice setting. The Outreach Care in Shortened Periodontitis Therapy (§22a SGB V) provision targets vulnerable groups who may face challenges in maintaining oral hygiene or accessing dental services, such as individuals requiring long‐term care or those with disabilities or care needs. For these patients, a modified and shortened periodontal treatment approach is available, emphasizing a barrier‐free and less bureaucratic pathway to care. In contrast, the Systematic Periodontitis Therapy (PAR Guidelines of the GKV) provides a structured framework for the systematic treatment of periodontitis within the statutory health insurance system for the general population. This approach involves comprehensive diagnostics, treatment planning, and a sequence of therapeutic measures, including anti‐infective therapy (AIT) and supportive periodontal care (German: “Unterstützende Parodontitistherapie [UPT]”). In contrast, §22a SGB V focuses on vulnerable groups with specific needs, such as individuals requiring long‐term care or those with disabilities. The shortened outreach periodontal therapy approach under §22a SGB V is based on two‐point pocket measurement without radiological diagnostics, and includes subgingival instrumentation of all pockets with a probing depth of 4 mm or more (regardless of whether there is bleeding or not), followed by a fixed interval for UPT sessions. Additionally, outreach care under §22a SGB V is often delivered through mobile or domiciliary services to accommodate patients with mobility or cooperation challenges, while systematic therapy following the PAR guidelines is typically carried out in standard outpatient dental practices. The current literature offers limited insights into the feasibility and clinical outcomes of providing periodontal treatment in outreach settings for this vulnerable population. We aimed to examine the clinical effectiveness of the shortened periodontal therapy approach according to §22a SGB V in an outreach setting compared to no periodontal therapy over 3 months, evaluating plaque, gingivitis, and probing depth among nursing home residents diagnosed with periodontitis.

## 2 MATERIALS AND METHODS

The study received ethical approval from the local ethics review board of the University of Cologne, Cologne, Germany (22‐1219), and was registered in the German Clinical Trials Register (DRKS) prior to patient enrollment (registration number DRKS00029392, date 09‐13‐2022). All procedures complied with the ethical standards of the institutional research committee, the 1964 Helsinki Declaration, and subsequent amendments or comparable ethical standards. The study adhered to the CONSORT guidelines. Informed consent was obtained from all participants or their legal guardians. For ethical reasons, the control group was offered the shortened outreach periodontal therapy approach after a follow‐up of 3 months (not part of our study). Therefore, all study participants received treatment in accordance with the guidelines applicable in Germany within a time frame that aligns with the standards set by statutory health insurance.

### 2.1 Study population

Nursing home residents with periodontitis requiring outreach dental care according to §22a in Germany were approached to participate; those who agreed (or their legal advisors) and provided written informed consent were included (October 2022 to March 2024). The study dentist (DiB) was the cooperating dental practitioner responsible for the dental care of all residents. Participants had regular medical and dental health institutional insurance. All individuals were aged > 65 years, nursing home residents, had no antibiotic therapy within the past 6 months, and were in need of periodontal treatment classified according to the PSI (Periodontal Screening Index, analogue to the CPITN).^7^ A minimal probing depth of 4 mm defined the need for periodontal therapy, as specified in the treatment guidelines in Germany within which the therapy was performed.^25^ Exclusion criteria included life‐threatening conditions indicating imminent death or not meeting the statutory health insurance requirements for periodontal therapy (e.g., only a general anesthetic was feasible).

### 2.2 Sample size

For scaling and root planing and 1 month following treatment Nakajima et al. observed a reduction in BOP from 100% (19/19) to 42% (8/19).^26^ We expected to see a similar difference (corresponding to a Cohen's d of 1.3 [$\approx \left(1-0.42\right)/\sqrt{\bar{p}\cdot \left(1-\bar{p}\right)}$ with $\bar{p}=\left(1+0.42\right)/2$] between groups. Thus, cautiously assuming a true effect size of *d* = 1.0 between groups, the unpaired *t*‐test requires 17 patients per group for 80% power at a two‐sided significance level of 5% (Stata/SE 17.0, StataCorp LLC, College Station, TX, USA). Allowing for 5% dropouts per group, one additional participant was added, resulting in 18 participants per group, total 36 randomized individuals.

### 2.3 Study design

Personal health measures (care dependency level (as per German law), cognitive status, prescribed medications, systemic diseases) were obtained from medical files of the nursing homes. Participants were randomized to intervention or control, using a randomization list created prior to the start of the study. When a patient was recruited, the study dentist contacted the study center to determine the allocation group.

Patients in the intervention group received the shortened outreach periodontal therapy approach (§22a SGB V) in the nursing home, according to German statutory health insurance requirements, and were visited after 3 months for the follow‐up appointment. The control group received a baseline examination and follow‐up examination after 3 months, without further dental treatment. For ethical reasons, the control group was offered periodontal therapy after follow‐up (not part of our study). As part of the shortened outreach periodontal therapy, the study participants received oral hygiene optimization in accordance with the guidelines mandated by German statutory health insurance.^27^ As part of this study, the participants and nursing staff were briefed on the necessary aspects of oral hygiene optimization before the treatment session. This oral hygiene briefing included both documentation of current care status and a summary of required improvements, as documented in the German care documentation protocol, which is designed to inform both the care recipients and the nursing staff.^28^ All nursing home staff in the nursing homes received annual oral care tutorials from the cooperating dentist.

### 2.4 Periodontal therapy: Intervention group appointments

The therapy steps followed the Adaptation of the European Federation of Periodontology S3 clinical practice guideline^29^ and its adaptation to the German statutory health system. In this outreach care approach, shortened periodontitis therapy in the nursing home involved the following steps: (1) pre‐baseline: examination, advice, screening with a PSI probe, identification of periodontal treatment needs, anamnesis, consent from participants or legal guardians, clarification of study participation, randomization; (2) baseline appointment: examination of bleeding on probing (BOP), plaque index (PI), and probing pocket dept (PPD)—instrumentation included subgingival instrumentation, polishing of tooth surfaces, and disinfection therapy with chlorhexidine (CHX; rinsing with CHX after subgingival instrumentation may be performed especially in vulnerable populations^29^); (3) follow‐up examinations: the first follow‐up after 7 days assessed the PI, while the second follow‐up after 3 months replicated the baseline examination.

### 2.5 Intervention procedures

At baseline, inclusion criteria were checked. For the periodontal assessment, BOP, PI, and PPD full‐mouth measurement was performed as a two‐point measurement mesial and distal, as defined in the German treatment guideline,^25^ using a periodontal probe*.

Subgingival instrumentation was performed using hand instruments, including curettes† and a machine‐driven scaler‡. The treatment included polishing tooth surfaces and pocket irrigation§. All procedures were conducted in the nursing home using a mobile unit‖. Additional products included polishing paste¶, polishing brushes with nylon bristles#, and interdental brushes of various individualized diameters**.

### 2.6 Endpoints

The primary endpoint was BOP. Secondary endpoints included the PI, mean and maximal PPD, and a self‐developed assessment of cooperation during instrumentation after the sessions by the study dentist for AIT, and the definition of number of necessary AIT appointments, according to an oral care form used in Germany for those needing care.^28^ The time required for the AIT procedure (min) was also recorded. BOP, PI, and PPD were measured at the beginning of each examination session.^30^, ^31^, ^32^ Further information regarding Care need classification in Germany is provided in Supplement 1in the online *Journal*.

### 2.7 Statistical analysis

Descriptive statistics (mean, standard deviation, range) were calculated for BOP, PI, and PPD for each group and examination date. Inter‐individual differences between groups were determined using the Mann–Whitney *U* test; intra‐individual differences (∆) within the therapeutic course were evaluated using the Wilcoxon signed‐rank test. A tooth‐type specific subgroup analysis was performed. Specific information is provided in Supplement 1. Statistical analyses were done using SPSS Statistics 28.0 software††.

## 3 RESULTS

**FIGURE 1:**
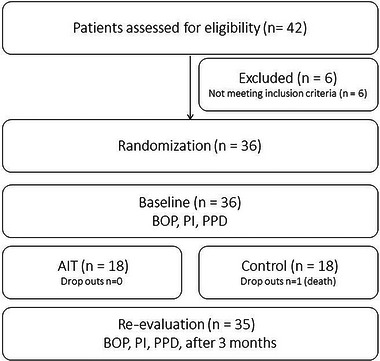
Study flowchart. AIT, anti‐infective therapy; BOP, bleeding on probing; PI, plaque index; PPD, pocket probing depth.

Figure 1 shows the study flow chart. Of the 42 eligible nursing home residents approached to participate, 36 agreed. Six people did not participate: one passed away shortly after being contacted, two siblings relocated to a different care facility, two legal guardians declined to provide consent without specifying a reason, and one participant was undergoing continuous antibiotic treatment.

Thus, 36 nursing home residents were included in the study (mean age 85 ± 6 years; 27 (75%) female. There were no significant differences in demographic characteristics between intervention and control groups (Table 1). Patients were prescribed a mean of 11 ± 4 medications/day, only one patient was a smoker, and dementia was (neurologist) diagnosed in 21 (58%) patients. Systemic diseases are shown in Table 2. The mean care level was 3.5 ± 0.8, with the intervention group tending toward higher levels of care.

**TABLE 1:** Patient and clinical characteristics.

	All (*n* = 36)	Intervention (*n* = 18)	Control (*n* = 18)	
Characteristics	Mean ± standard deviation (min.−max.)			*p*‐value
Age (years)	84.8 ± 6.2	85.2 ± 5.6 (73–93)	84.5 ± 6.9 (69–95)	0.719^*^
Active pharmaceutical ingredients^a^	11.1 ± 4	11.4 ± 3.4 (5–19)	10.8 ± 4.6 (6–20)	0.628^*^
Care level	3.5 ± 0.8	3.8 ± 0.8 (3–5)	3.2 ± 0.8 (2–5)	0.085^*^
No. of teeth	17 ± 7.5	17.4 ± 7.3 (6–30)	16.6 ± 7.9 (6–28)	0.767^*^
No. of teeth with periodontal treatment need	15.6 ± 7.0	15.6 ± 6.8 (6–27)	15.6 ± 7.4 (3–27)	1.000^*^
% of teeth with periodontal treatment need	94.7 ± 10.3	96.9 ± 4.8 (86–100)	92.5 ± 13.6 (50–100)	0.628^*^

	*n* (%)			
Sex				
Female	27 (75)	13 (72.2)	14 (77.8)	0.700^b^
Male	9 (25)	5 (27.8)	4 (22.2)	
Dementia diagnosis				
Yes	21 (58.3)	11 (61.1)	10 (55.6)	0.735^b^
No	15 (41,7)	7 (38.9)	8 (44.4)	
Oral hygiene				
Independently	20 (55.6)	11 (61.1)	9 (50.0)	0.531^b^
Partial takeover	15 (41.7)	7 (38.9)	8 (44.4)	
Full takeover by nursing staff	1 (2.8)	0	1 (5.6)	
Cooperation				
Full cooperation	18 (50.0)	9 (50.0)	9 (50.0)	0.871^b^
Sufficient cooperation	13 (36.1)	6 (33.3)	7 (38.9)	
Deficient cooperation	5 (13.9)	3 (16.7)	2 (11.1)	

^a^ Active pharmaceutical ingredients (APIs): the active components in a drug that produce the intended therapeutic effect.

^b^ Pearson Chi‐Square test.

* Mann–Whitney *U* test.

**TABLE 2:** Prevalence of diseases among patients in the intervention and control groups.

No.	Disease	Intervention group (*n* = 18), *n* (%)	Control group (*n* = 18), *n* (%)
1	Cardiovascular diseases	15 (83.3)	11 (61.1)
2	Cancer	1 (5.6)	0 (0.0)
3	Chronic lung diseases (chronic obstructive pulmonary disease, pulmonary embolism)	4 (22.2)	9 (50.0)
4	Musculoskeletal disorders	7 (38.9)	2 (11.1)
5	Diabetes mellitus	5 (27.8)	3 (16.7)
6	Dementia disorders	10 (55.6)	9 (50.0)
7	Depression/other psychiatric diseases	7 (38.9)	3 (16.7)
8	Neurological/neurodegenerative disorders	7 (38.9)	1 (5.6)
9	Kidney diseases	7 (38.9)	4 (22.2)
10	Eye diseases (e.g., age‐related macular degeneration, glaucoma)	3 (16.7)	2 (11.1)
11	Gastrointestinal diseases	3 (16.7)	7 (38.9)
12	Metabolic disorders (e.g., thyroid disorders, lipid metabolism disorders, hyper/hypolipidemia, hypercholesterolemia)	12 (66.7)	8 (44.4)
13	Obesity	3 (16.7)	1 (5.6)

### 3.1 Baseline oral health characteristics

Overall, 20 (55.6%) patients were able to conduct their own oral hygiene routines according to assessment by the nursing staff, 15 (41.7%) required partial assistance, and one (2.8%) relied entirely on nursing staff for oral hygiene maintenance. Participants had a mean of 17 ± 7.5 natural teeth. In the intervention group, the mean BOP was 45 ± 20, PI was 1 ± 0.4, and PPD was 4.5 ± 0.5 mm. In the control group, the mean BOP was 33 ± 25, PI was 0.8 ± 0.4, and PPD was 4.3 ± 0.5 mm (Table 3). Comparing baseline measures between intervention and control, no differences were found (BOP *p *= 0.059, PI *p *= 0.096, mean PPD *p *= 0.106, max PPD 0.160, Mann–Whitney *U* test).

**TABLE 3:** Periodontal measures before and 3 months after anti‐infective therapy.

Clinical endpoint	Intervention (*n* = 18)	Control (*n* = 18)	*p*‐value^*^
Percentages index (mm)	Mean ± standard deviation (min.−max.)		Cohen's *d* 95% (CI)
**Bleeding on probing (%)**			
Baseline	45 ± 20 (0–94)	33 ± 25 (0–86)	
FU‐1 (3 months)	35 ± 16 (8–73)	38 ± 25 (0–85)^a^	
***p*‐value** ^b^	0.088	0.309	
∆ BOP (BL to FU‐1)	−10 ± 26 (−67–35)	5 ± 16 (−28–38)^a^	**0.041** *d = −0.70* *(−1.40* to *0.00)*
**Plaque index**			
Baseline	1.0 ± 0.4 (0.2–1.6)	0.8 ± 0.4 (0.1–1.6)	
FU‐1 (3 months)	0.7 ± 0.4 (0.2–1.2)	0.7 ± 0.4 (0.1–1.4)^a^	
***p*‐value** ^b^	**0.010**	0.393	
∆ Plaque (FU‐1 to BL)	−0.3 ± 0.4 (−0.9–0.9)	−0.1 ± −0.3 (−0.6–0.4)^a^	0.09 ^a^ *d* = − 0.56 (−1.24 to 0.11)
**Mean probing pocket depth (mm)**			
Baseline	4.5 ± 0.5 (3.8–5.6)	4.2 ± 0.6 (3.6–5.9)	
FU‐1 (3 months)	4.0 ± 0.5 (2.8–5.2)	4.3 ± 0.5 (3.6–5.1)^a^	
***p*‐value** ^b^	**0.004**	0.136	
∆ mean PPD (FU‐1 to BL)	−0.5 ± 0.5 (−1.5–0.2)	0.1 ± 0.4 (−0.8–0.6)^a^	**<0.001** *d = *−*1.32* (−*2.05* to *‐0.59)*
**Maximum probing pocket depth (mm)**			
Baseline	6.0 ± 1.0 (4–8)	5.5 ± 0.6 (5–7)	
FU‐1 (3 months)	5.4 ± 0.8 (4–7)	5.6 ± 0.8 (5–7)^a^	
***p*‐value** ^b^	**0.008**	0.317	
∆ max PPD (FU‐1 to BL)	−0.6 ± 0.7 (−2–1)	0.1 ± 0.8 (−1–2)^a^	**0.026** *d = *−*0.93* (−*1.63* to *‐0.23)*

*Note*: All bold values represent statistical significance.

Abbreviations: BL, baseline; BOP, bleeding on probing; CI, confidence interval; FU, follow‐up; PPD, pocket probing depth.

^a^ *N* = 17.

^b^ Wilcoxon signed‐rank test.

* Mann‐Whitney *U* test and *t*‐test.

### 3.2 Anti‐infective therapy

Table 3 shows the clinical measures 3 months after shortened outreach periodontal therapy. At 3 months, BOP decreased in the intervention group (to 35 ± 16) and increased slightly in the control group (to 38 ± 25), with no significant within‐group differences (intervention: *p *= 0.088; controls: *p *= 0.309). However, when comparing the intervention effect (ΔBOP −10 ± 26) with controls (ΔBOP 5 ± 16), a significant difference between groups was observed (*p *= 0.041). Nevertheless, the effect size analysis revealed a Cohen's d of −0.70 with a 95% confidence interval (CI) of [−1.40 to 0.00], indicating that while the effect was statistically significant, its robustness remains uncertain.

After 7 days, there was a significant reduction in the PI (to 0.5 ± 0.3; *p *< 0.001) in the intervention group. After 3 months, PI values were similar in both groups (mean 0.7 ± 0.4). The intervention effect (ΔPlaque −0.3 ± 0.4) compared to controls (ΔPlaque ‐0.1 ± 0.3) did not reach statistical significance (*p *= 0.09). The effect size for this comparison was *d* = −0.56 (95% CI [−1.24 to –0.11]), suggesting a moderate but uncertain effect.

At 3 months, the mean PPD decreased significantly in the intervention group (to 4.0 ± 0.5 mm; *p *= 0.005), while it remained stable in the control group (4.3 ± 0.4 mm). The intervention effect was significantly different between groups (*p *< 0.001) (corrected for multiple testing (i.e., 12 comparisons), the group difference in mean PPD would still reach statistical significance (*p_adj _* < 0.012)), with a corresponding effect size of *d* = −1.32 (95% CI [−2.05 to −0.59]), indicating a strong treatment effect. The maximum PPD also decreased significantly in the intervention group (Δ max PPD ‐0.6 ± 0.7 mm; *p *= 0.026), with a Cohen's d of −0.93 (95% CI [−1.63 to −0.23]), supporting a clinically relevant reduction in deep periodontal pockets.

In addition, the analysis of PPD revealed a significant improvement in the intervention group (Table 4). The percentage of healthy periodontal pockets (1–3 mm) increased significantly from baseline (8.2 ± 10.3%) to the three‐month follow‐up (25.1% ± 28.4%; *p *= 0.036). Additionally, there was a trend toward a reduction in deep periodontal pockets (≥6 mm), with a mean decrease of 8.0 ± 14.5% (*p *= 0.016). While the percentage of moderate pockets (4‐5 mm) showed a slight decrease in the intervention group (−8.8% ± 30.0), this change did not reach statistical significance (*p *= 0.134). These findings indicate a beneficial effect of the intervention on periodontal health by promoting a shift toward shallower pocket depths.

**TABLE 4:** Analysis of periodontal pocket depths.

	Intervention (*n* = 18)	Control (*n* = 17)	
	Mean ± standard deviation (min.−max.)		*p*‐value^*^
**Percentage of PPD healthy (1–3 mm)**			
Baseline	8.2 ± 10.3 (0–32)	16.1 ± 18.8 (0–50)	
FU‐1 (3 months)	25.1 ± 28.4 (0–91)	9.2 ± 13.1 (0–40)	
***p*‐value** ^a^	**0.036**	0.075	
Δ percentage PPD healthy (BL to FU‐1)	16.8 ± 28.9 (−16–83)	−6.9 ± 16.1 (−48–21)	**0.019**
**Percentage of PPD moderate (4–5 mm)**			
Baseline	79.4 ± 14.8 (50–95)	76.2 ± 21.0 (37–100)	
FU‐1 (3 months)	70.6 ± 26.1 (9–100)	83.6 ± 11.0 (60–100)	
***p*‐value** ^a^	0.296	0.266	
Δ percentage PPD moderate (BL to FU‐1)	−8.8 ± 30.0 (−77–50)	7.4 ± 21.6 (−21–53)	0.134
**Percentage of PPD deep (6 mm and more)**			
Baseline	12.4 ± 15.2 (0–50)	7.6 ± 15.4 (0–64)	
FU‐1 (3 months)	4.4 ± 8.4 (0–33)	7.2 ± 8.2 (0–23)	
***p*‐value** ^a^	**0.016**	0.646	
Δ percentage PPD deep (BL to FU‐1)	−8.0 ± 14.5 (−50–4)	−0.4 ± 12.5 (−41–19)	0.083

*Note*: All bold values represent statistical significance.

Abbreviations: BL, baseline; FU, follow‐up; PPD, probing pocket depth.

^a^ Wilcoxon signed‐rank test.

* Mann‐Whitney *U* test.

### 3.3 Duration and number of therapy sessions and patient cooperation

There was no difference in cooperation during the baseline examination between groups, respectively, as assessed by the treating dentist (*p *= 0.822). Full cooperation was shown by nine (50%) patients in each group, sufficient cooperation by six (33.3%) and seven (38.9%) patients, with deficient cooperation in three (16.7%) and two (11.1%) patients. Deficient cooperation may be interpreted to mean that a minimal but significantly shortened intervention was anticipated, as it was expected that therapy would need to be interrupted multiple times due to challenging compliance. In the intervention group, 13 (72.2%) patients needed one appointment for the intervention session, while the others received two appointments. The mean duration of subgingival instrumentation at each appointment was 14 ± 5.4 (range 7–30) min.

### 3.4 Site‐dependence as influencing variable

**FIGURE 2:**
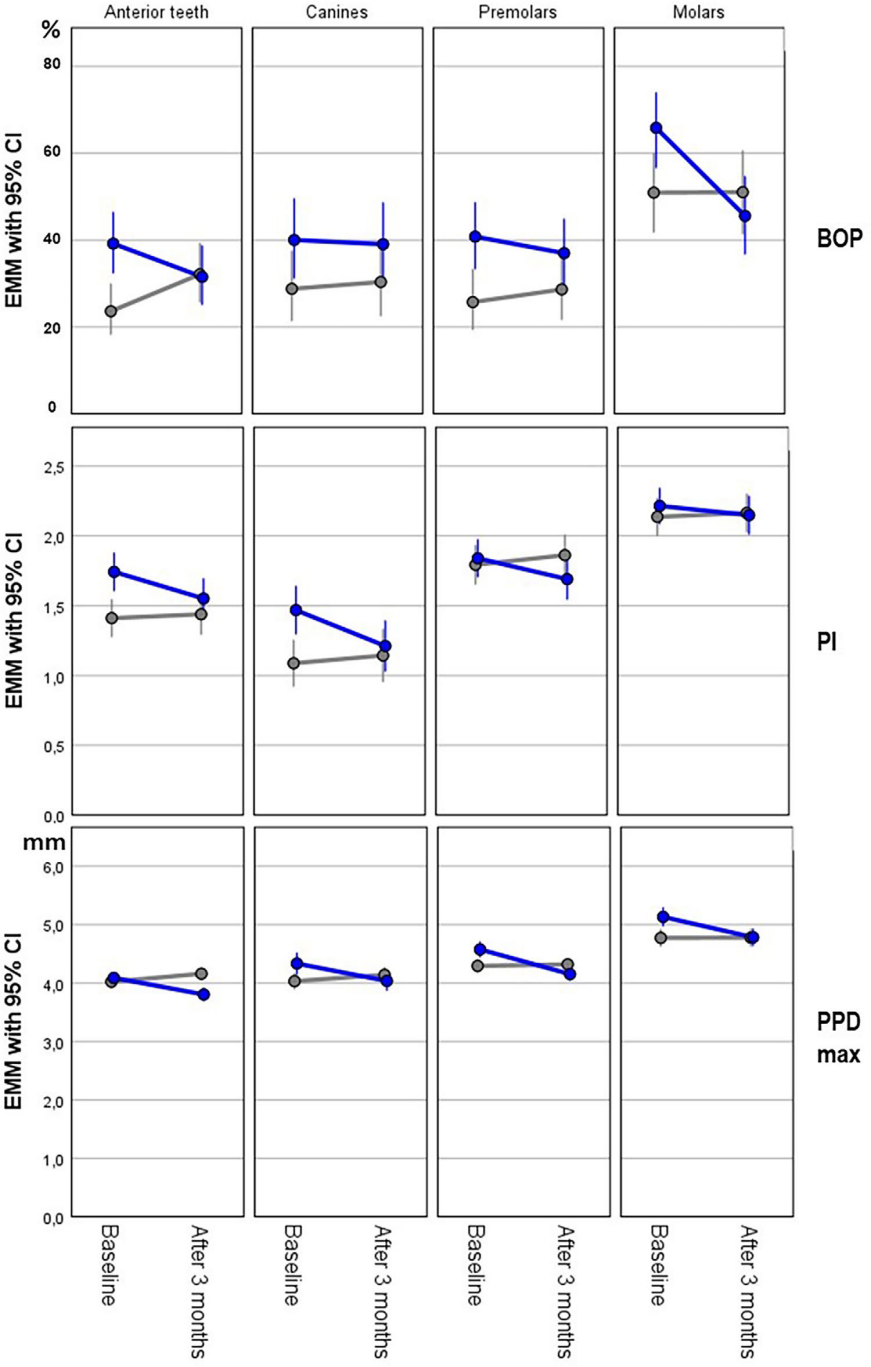
Estimated marginal means (EMMs) with 95% confidence interval (CI) for different tooth groups, from baseline to follow‐up after 3 months. BOP, bleeding on probing; PI, plaque index; PPD, pocket probing depth.

Analysis of independent variables suggested significant variables for the dependent variables BOP (tooth group, *p *< 0.001; d/m localization, *p *= 0.030), PI (tooth group, *p *< 0.001; o/v localization, *p *= 0.037; upper or lower jaw, *p *< 0.001), and PPD (tooth group, *p *< 0.001, d/m localization, *p *= 0.021; upper or lower jaw, *p *= 0.001). However, most disappeared when tested for the treatment group*time*independent variable. Treatment group remained a significant influencing factor for BOP (*p *= 0.002) and for PPD (*p *< 0.001) when tested for treatment group*time. For the dependent variable PI, an influencing factor was o/v localization of plaque (*p *= 0.030) when testing for treatment group*time*o/v. Figure 2 shows the longitudinal course from baseline to follow‐up as a result of the mixed‐model analysis.

## 4 DISCUSSION

As expected, our nursing home residents had poor oral hygiene and periodontitis at baseline, a finding that is well described in the literature.^5^ Site‐specifically, BOP, PI, and PD have been shown in other populations to be higher in the molars than in front teeth.^33^ We observed improvements in clinical periodontal measures compared to controls, including reduced gingival inflammation and probing depths in between‐group comparison and longitudinal improvements in secondary (mean and max PPD) outcome measures after 3 months in the intervention group. This is remarkable, considering the isolated AIT (with no changes in oral hygiene as part of the intervention), the obvious oral hygiene deficits, multimorbidity, and care needs in this special patient population, and the fact that the groups had limitations in terms of the patients’ ability to cooperate (assessed by the treating dentist) and the organizational conditions associated with outreach dental care settings. It should be emphasized that after 3 months, outcomes were stable or improved in the intervention group but stable or deteriorating in controls. For all endpoints, treatment (intervention or control) was a significant influencing factor for treatment success over time; for PI, o/v plaque location influenced treatment effects. Many other influencing factors were tested (e.g., tooth group, m/d location, age, upper or lower jaw), but all failed to remain significant when tested for treatment group over time.

Remarkably, the therapeutic effect with intervention was achieved in 14 min on average for an AIT session, which is less than the duration of a corresponding session in a dental practice, and was mainly because participants could not cooperate for longer. Organizational framework conditions, such as inadequate light or treatment at the patient's bedside or in the common room, also posed hurdles. However, it should be noted that the number of teeth to be treated was significantly lower than in younger populations. Further investigation would determine the clinical effects if more time could be allocated for such treatments or if all participants were included (even those classified as uncooperative), under optimized organizational conditions. Yet even if conditions were optimized, the outcomes would have been rated as insufficient in a practice setting containing younger, healthier patients.

In our study, the intervention was statistically significantly successful—but how should these results be interpreted from a clinical perspective? For example, the statistically significant improvement in the mean PD is only marginal from a clinical perspective—especially since it does not represent a satisfactory result compared to healthier, younger patients, where healthy teeth are the treatment target. The same applies to the improvement in oral inflammation, as the BOP levels achieved were unsatisfactory. The fact that subgingival instrumentation in other populations is fundamentally capable of contributing to a greater clinical improvement in oral inflammation and PPD has been shown in a large number of studies.^29^, ^34^, ^35^, ^36^ However, our results should not be compared with those seen after comprehensive, systematic periodontal therapy, which often starts with professional tooth cleaning, is accompanied by extensive oral hygiene training, and then leads to regular stabilizing recall sessions. We only report results 3 months after, usually a single treatment session with supra‐ and subgingival instrumentation

As in many other studies, our study population demonstrates that oral hygiene in inpatient care facilities is inadequate, likely due to various factors (e.g., organizational conditions, staff turnover, vacancies, lack of dental training in nursing, little interest from elderly people themselves)^5^, ^37^, ^38^ and treatment effects failed to reach significance when compared between groups although standardized oral hygiene instructions were given to participants and nursing staff at baseline. Possibly more than in younger populations, additionally addressing oral hygiene during periodontal therapy an even higher frequency will obviously lead to better results. Various concepts for additionally improving oral hygiene have already been described; these should be combined with systematic periodontal therapy and scientifically investigated. Hypothetically, more positive effects on oral hygiene measures in our study would probably have led to stronger effects on oral inflammation, plaque levels, and reduced probing depths; these should be investigated in future studies.^39^, ^40^, ^41^ Treatment effects achieved in the intervention group were based on supra‐ and subgingival instrumentation only, with no further oral hygiene interventions. Thus, there is a clear justification for systematic periodontal treatment, including improved oral hygiene, in this patient group.

Increasing evidence (especially in younger populations) describes a bidirectional link between periodontitis and various (mainly inflammatory) diseases; it is plausible that these associations are also true in older populations. In addition, there is academic consensus that a healthy oral cavity contributes to systemic health and well‐being, especially in old age.^42^ Immunosenescence and inflammaging certainly contribute to the high periodontal disease burden in our population.^43^ Age‐related changes in neutrophils, macrophages, and T cells, for example, result in inflammaging and impaired immune function (immunosenescence) and an inadequate immune response to oral invading pathogens, leading to their chronic persistence within the body and a shift of the periodontal treatment burden to the later decades of life.^2^, ^44^ At this point in time, it is not well described how an improved clinical situation will affect systemic inflammation and other chronic diseases in this particular population, which should be the subject of future investigations. Yet systemic diseases certainly contribute to the therapeutic outcomes resulting from shortened outreach periodontal therapy—be it via altered immune responses, current medications, or quite simply through the altered ability to perform good oral hygiene.

### 4.1 Limitations

Our study has limitations. A particular strength is the randomized, controlled analysis of a rarely investigated, guideline‐compliant treatment course in on‐site outreach care in inpatient care facilities. A limitation is that the periodontal therapy was carried out by one dentist. However, we carefully selected the dentist, who was calibrated and has specialist dental standards for geriatric dentistry; thus, we assume that the therapy was carried out in the best possible way. The chosen sample size of 36 participants per group may appear low in terms of the external validity of the results. However, an a priori sample size calculation was conducted, taking potential dropouts into account. The results demonstrated that even with this seemingly small sample size, the intervention achieved significant effect sizes, and group differences became apparent. No professional teeth cleaning was carried out to standardize oral hygiene measures at baseline. Nevertheless, our study shows a real‐life scenario in an inpatient care facility; the baseline findings correspond to corresponding populations from the literature. Accordingly, some of the measured values appear to be too low in the population, such as a possible BOP of 0 in the range documented in Table 3. Nevertheless, these are the documented measures. We explain this by the fact that in a difficult setting the examination conditions may not be optimal at all times, so that these minimum values may have to be interpreted carefully.

## 5 CONCLUSIONS

In this highly vulnerable population of nursing home residents—who had limited everyday competence and/or care needs, and who were sometimes not fully cooperative— shortened outreach periodontal therapy with subgingival instrumentation was able to improve clinical periodontal measures such as gingival inflammation and periodontal probing depths. Plaque levels were longitudinally reduced, although statistical significance in treatment effects between groups was not reached. The clinical parameters investigated were highest in the molar region. Overall, the changes in clinical parameters must be considered modest, likely due to the oral hygiene situation in this vulnerable population. The absence of significant improvements in BOP indicates that oral hygiene measures were insufficient to sustain the treatment benefits over time. Nevertheless, given the demonstrated short‐term effectiveness and the critical role of periodontal health in overall well‐being for this vulnerable population, implementing shortened outreach periodontal therapy in nursing homes remains a justified and necessary approach. However, there is a need for further investigation into how these accompanying measures, particularly oral hygiene optimization, can be enhanced to ensure such interventions have a lasting effect.

## AUTHOR CONTRIBUTIONS

All authors have made substantial contributions to conception and design of the study. Anna G. Barbe, Dalia Beck, Dirk Bleiel, Martin Hellmich, and Sonja H. M. Derman have been involved in data collection and data analysis. Anna G. Barbe, Dirk Bleiel, Max von Kohout, Martin Hellmich, and Sonja H. M. Derman have been involved in data interpretation. Anna G. Barbe, Martin Hellmich, Dirk Bleiel, and Sonja H. M. Derman have been involved in drafting the manuscript and revising it critically and have given final approval of the version to be published. All authors agree to be accountable for all aspects of the work in ensuring that questions related to the accuracy or integrity of any part of the work are appropriately investigated and resolved.

## CONFLICT OF INTEREST STATEMENT

The authors declare no conflicts of interest.

## ETHICS STATEMENT

The study received ethical approval from the local ethics review board of the University of Cologne, Cologne, Germany (22‐1219). All procedures complied with the ethical standards of the institutional research committee, the 1964 Helsinki Declaration, and subsequent amendments or comparable ethical standards. Informed consent was obtained from all participants or their legal guardians.

## Supporting information



Supporting Information

## Data Availability

The data that support the findings of this study are available from the corresponding author upon reasonable request.

## Footnotes

* Carl Martin probe, Henry Schein Dental Deutschland GmbH, Langen, Germany

† Gracey and Universal curettes, Stoma Dentalsysteme GmbH & Co KG, Emmingen‐Liptingen, Germany

‡ Piezon Master 250, EMS S.A., Munich, Germany

§ Chlorhexamed Rinse forte 0.2%, GlaxoSmithKline Consumer Healthcare, London, United Kingdom

‖ Viva Ace, NSK Europe GmbH, Eschborn, Germany

¶ Cleanic polishing paste, Kerr GMBH, Herzogenrath, Germany

# Komet 9533M.000.060 VPE 100, Komet Dental, Gebr. Brasseler GmbH & Co. KG, Lemgo, Germany

** TePe 1805/2, TePe D‐A‐CH GmbH, Hamburg, Germany

†† IBM Corp., Armonk, NY, USA
