# Supplementary material for: Shortened outreach periodontal therapy in nursing home residents with periodontitis: A randomized controlled trial
Source: J Periodontol. 2025 Dec 26;97(6):1170–82. doi: 10.1002/jper.70041 (PMC13350520; doi:10.1002/jper.70041)
Supplement: Supplementary file 1 — Supporting Information [file JPER-97-1170-s001.docx]

**Care need.** Germany’s long-term care system employs a structured classification to assess and address individual care needs, known as care degrees (Pflegegrade).^1^ Introduced in 2017, this system comprises five distinct levels: a) Care Degree 1: Slight impairment of independence; b) Care Degree 2: Considerable impairment of independence; c) Care Degree 3: Severe impairment of independence; d) Care Degree 4: Most severe impairment of independence; e) Care Degree 5: Most severe impairment of independence with special requirements for nursing care. These degrees are determined through a standardized assessment focusing on the individual’s capabilities and limitations across various domains, including mobility, cognitive and communicative abilities, behaviors, self-care, and management of daily life tasks. The assigned care degree dictates the scope and extent of benefits provided by the long-term care insurance, encompassing services like home care, day and night care, short-term residential care, and full residential care.

**Statistical analysis.** Descriptive statistics (mean, standard deviation, range) were calculated for BOP, PI, and PPD for each group and examination date. Inter-individual differences between groups were determined using the Mann-Whitney *U-* test; intra-individual differences (∆) within the therapeutic course were evaluated using the Wilcoxon signed-rank test. A tooth-type specific subgroup analysis was performed. Therefore, the outcome variables BOP, PI and PD were analyzed using generalized linear models for repeated measures (within subject variables: Treatment, with covariates age and number of previous illnesses; between subject variables: Time (from baseline to 3-month follow-up), Group (incisors, canines, premolars, molars), Jaw (upper or lower), ‘d/m’ (distal or mesial), ‘o/v’ (oral or vestibular). To investigate the influence of the intervention on specific locations, the following interactions were specified: Treatment*Time, Group*Time, Treatment*Time*Group, Treatment*Time*o/v, Treatment*Time*Jaw, Treatment*Time*d/m. Based on these analyses, estimated marginal means (EMMs) were calculated and compared pairwise (difference with 95% confidence interval and p-value). Given the differing scales of the endpoints, a binary outcome with a logit link function was specified for the BOP model. For the PI and PPD models, a continuous Gaussian-distributed endpoint with an identity link function was used. Profile plots with 95% confidence intervals were created for the marginal means over time, group, and treatment. P-values p<0.05 were considered statistically significant and not corrected for multiple testing to retain statistical power. Specifically, only the primary endpoint “bleeding on probe (BOP)” was tested “confirmatory” for a between-group difference in location. All other endpoints were considered “secondary” and evaluated without strong control of the type-I error. However, the corresponding p-values may be Bonferroni-corrected for multiple testing by multiplying them with the number of associated tests, e.g. 12, see Table 3 in the manuscript.

**Reference**

1. Federal Ministry for Health: Department of public relations and publications. Long-Term Care Guide. Everything you need to know about long-term care. [serial online]. 2020. Available from: bundesgesundheitsministerium.de/fileadmin/Dateien/5_Publikationen/Pflege/Broschueren/200320_BMG_Ratgeber-Pflege_DINA5_ENG_bf.pdf. Accessed 24 March 2025.
